# Exploring Adults’ Experiences with Tirzepatide for Weight Loss: A Mixed-Methods Study

**DOI:** 10.3390/healthcare13233102

**Published:** 2025-11-28

**Authors:** Shukri Adam, Fatma M. Ibrahim, Eman Abdelaziz Ahmed Dabou, Sneha Pitre, Rania Aiman, Shimaa AbdelSamad

**Affiliations:** 1 RAK College of Nursing, RAK Medical and Health Science University, RAS Al-Khaimah P.O. Box 11172, United Arab Emirates; 2 Faculty of Nursing, Mansoura University, Mansoura 35516, Egypt; 3 Faculty of Nursing, Jordan University of Science and Technology, Irbid 22110, Jordan; 4 Faculty of Nursing, Alexandria University, Alexendria 21568, Egypt

**Keywords:** primary care, community health, tirzepatide, obesity, mixed-methods, self-efficacy, patient-reported outcomes

## Abstract

**Highlights:**

**What are the main findings?**

- Among 120 adults (mean age 42 ± 13 years; 50.8% male), 91.7% reported weight loss after initiating tirzepatide.
- Average Weight Efficacy Lifestyle (WEL) total score was 91 ± 34 (0–180 scale); higher WEL was observed in females, employed participants, those with coverage, and early in treatment.
- Interviews (n = 15) described high satisfaction with weight loss, better sleep/energy/mood, and mostly mild/transient gastrointestinal effects.
- Findings support pairing pharmacotherapy with behavioral support and addressing affordability/coverage to sustain benefits.

**What is the implication of the main finding?**

- Findings support the integration of behavioral support alongside tirzepatide pharmacotherapy and highlight the importance of treatment affordability and coverage. Prospective longitudinal studies are warranted to assess the durability of self-efficacy and health outcomes and to test strategies that optimize adherence and equitable access.

**Abstract:**

**Background:** Obesity confers substantial cardiometabolic risk. Tirzepatide, a once-weekly dual GIP/GLP-1 receptor agonist, produces dose-dependent weight loss in trials, but real-world patient-reported experiences are under-described. We evaluated real-world self-efficacy and experiences with tirzepatide in community settings. **Methods:** Explanatory sequential mixed-methods study of adults aged 18–59 years using tirzepatide for weight management. We collected a quantitative survey (demographics; medication use; 20-item Weight Efficacy Lifestyle Questionnaire [WEL]) followed by purposive semi-structured interviews. Associations between WEL and participant characteristics were tested a priori (two-tailed α = 0.05). **Results:** Among 120 participants (50.8% male; mean age 42 ± 13 years), 91.7% reported weight loss and 85.8% had <6 months’ exposure. WEL total was 91 ± 34. Higher WEL was observed in females, employed participants, those with insurance coverage versus self-pay, during early months of therapy, and among those with prior weight-loss attempts (all *p* < 0.05). Interviews (n = 15) indicated high satisfaction, improved sleep/energy, mood, and confidence; gastrointestinal effects were usually mild/transient. **Interpretation:** In routine care, tirzepatide use was associated with high eating self-efficacy and positive patient-reported outcomes. Variation by coverage and duration suggests value in pairing pharmacotherapy with behavioral support and addressing affordability to sustain benefits.

## 1. Introduction

Obesity confers substantial cardiometabolic and psychosocial burden, and many adults achieve only modest, un-sustained loss with lifestyle measures alone [1]. Incretin-based pharmacotherapy has expanded treatment options, particularly glucagon-like peptide 1 (GLP 1) receptor agonists that enhance satiety, modulate glycaemia, and support weight reduction [2,3]. Tirzepatide, a once-weekly dual glucose-dependent insulinotropic polypeptide (GIP) and GLP 1 receptor agonist, reduces energy intake and improves glycemic control via complementary mechanisms [4,5].

Regulatory approvals include type 2 diabetes (U.S. FDA, May 2022) [6] and chronic weight management in adults with obesity or overweight plus a weight-related condition (U.S. FDA, November 2023) [7] as an adjunct to diet and activity. Randomized trials demonstrate dose-dependent weight loss (e.g., SURPASS-2 in type 2 diabetes; SURMOUNT-1/3 in obesity), with emerging evidence of greater weight reduction versus semaglutide in some populations [3,4,8].

Yet little is known about adults’ lived experiences in community settings, including perceived benefits, side effect trajectories, and eating self-efficacy in everyday contexts. Exit interviews from SURMOUNT-4 and digital-engagement studies offer insights but may not generalize to routine care [8,9]. Real-world perspectives are essential to contextualize trial findings, inform shared decision-making, and optimize long-term effectiveness. Consequently, the aim of the study was to characterize real-world patient-reported experiences with tirzepatide for weight management using an explanatory sequential mixed-methods design, quantifying eating self-efficacy and weight change and contextualizing these data with semi-structured interviews.

## 2. Materials and Methods

### 2.1. Study Design

We used an explanatory sequential mixed-methods design [10]: an initial quantitative survey followed by qualitative interviews to explain quantitative patterns. Integration occurred at interpretation through joint displays and meta-inferences. The study was approved by the Ministry of Health and Prevention Research Ethics Committee (MOHAP/REC/2025/14-2025-F-N). All participants provided written informed consent.

### 2.2. Setting and Participants

Adults were recruited from community locations in Ras Al Khaimah Emirate (malls, marketplaces, institutions, homes). Trained staff approached potential participants, confirmed eligibility, and obtained written informed consent prior to enrollment.

### 2.3. Eligibility Criteria

Inclusion: age 18–59 years; current or prior tirzepatide use for weight management; body mass index (BMI) ≥ 30 kg/m^2^ or ≥27 kg/m^2^ with ≥1 weight-related comorbidity (per indication). Exclusion: type 1 diabetes; contraindications per label (e.g., medullary thyroid carcinoma/MEN2); concomitant enrollment in another weight-loss trial; severe psychiatric/eating disorders impairing adherence or consent.

### 2.4. Sample Size and Sampling

A planned target of n = 267 (Raosoft: population ≈ 2000; confidence level 90%; margin of error 5%) was not reached due to time/resource constraints; recruitment during April 2025 yielded n = 120 complete quantitative cases. For the qualitative phase, a purposive subsample (n = 15) maximized variation by sex, treatment duration, weight-loss category, and WEL scores.

### 2.5. Outcomes

Primary outcomes: (a) self-reported percent weight loss since initiating tirzepatide (none, <5%, 5–10%, >10%); (b) eating self-efficacy (WEL total, 0–180). Secondary outcomes: item-level WEL responses; associations of WEL with demographics/clinical characteristics (age, sex, employment, insurance/coverage, BMI, duration of use, prior attempts).

### 2.6. Measures (Instruments)

Weight Efficacy Lifestyle Questionnaire (WEL): Total of 20 items rated 0 (“not confident”) to 9 (“very confident”); total 0–180 with higher scores indicating greater self-efficacy. Internal consistency for the total score is excellent (Nauck et al., 2022) [11]. We report total and item-level means.

Semi-structured interview guide: Interviews explored motivations, routines/self-management, side effects and dose titration, facilitators/barriers to adherence, and perceived changes in appetite, sleep, mood, activity, and overall satisfaction (Supplementary File S1).

### 2.7. Data Collection Procedures

Field team and steps to minimize bias: Data collection was conducted by two RN/PhD-trained researchers (approximate age range (25–36) years) who were not involved in participants’ clinical care. Interviewers followed standardized scripts, used neutral prompts, and conducted surveys/interviews privately to reduce social desirability and interviewer bias. Team members received protocol-specific training and practiced mock interviews; periodic debriefs were used to ensure fidelity and reflexivity.

### 2.8. Quantitative Analysis

Analyses used SPSS 29. Continuous variables are summarized as mean ± SD or median (IQR); categorical variables as n (%). Group differences for WEL used independent-samples *t*-tests (two groups) and one-way ANOVA (≥3 groups) with post hoc comparisons as appropriate. Assumptions were examined (Kolmogorov–Smirnov); we report 95% confidence intervals and two-tailed α = 0.05. Pearson correlations related WEL with age and BMI. Missing data (<5% per variable) were handled via listwise deletion.

### 2.9. Qualitative Analysis

Two analysts conducted reflexive thematic analysis (Braun & Clarke’s six phases). Coding discrepancies were resolved by discussion; an audit trail documented analytic decisions. A brief reflexive statement is provided in Supplementary File S1.

### 2.10. Mixed-Methods Integration

Quantitative results informed purposive sampling and interview prompts (connecting); we merged quantitative and qualitative data in joint displays to juxtapose WEL/weight-loss patterns with qualitative themes, generating integrative insights.

## 3. Results

### 3.1. Participant Characteristics

Interview subsample characteristics. The purposive interview subsample (n = 15) was selected to maximize variation in sex, treatment duration, self-reported weight-loss category, and WEL scores. A total of 120 adults (50.8% male; mean age 42 ± 13 years; 95% CI 39.7 to 44.3) were included. Most participants reported some weight loss (110/120; 91.7%; 95% CI 85.3% to 95.4%) after starting tirzepatide. Mean BMI at survey was 25.74 ± 3.96 kg/m^2^. Most had used tirzepatide for <6 months (85.8%). The mean WEL total score was 91 ± 34 (95% CI 84.9 to 97.1); Table 1 Continuous variables are presented as mean (standard deviation) with units; categorical variables as n (%).

### 3.2. Weight Efficacy Lifestyle (WEL) Items

Participants’ WEL item means generally clustered around 4–5 on a 0–9 scale, indicating moderate confidence across emotional and situational eating contexts, with notable strengths for resisting eating under social pressure, fatigue, and celebratory situations, and relatively lower confidence when highly palatable foods were available or under negative affect (Table 2).

### 3.3. Associations Between WEL Total and Participant Characteristics

Most participants reported some weight loss (110/120; 91.7%; 95% CI 85.3% to 95.4%) after starting tirzepatide. Mean BMI at survey was 25.74 ± 3.96 kg/m^2^. Most had used tirzepatide for <6 months (85.8%). The mean WEL total score was 91 ± 34 (95% CI 84.9 to 97.1). Table 3 key comparative estimates (differences in means, 95% CI): Female–Male (WEL): 7.0 (95% CI −5.1 to 19.1). Employed–Unemployed (WEL): 6.0 (95% CI: −4.9 to 16.9).

Exploratorily, given that 85.8% of participants were within the first 6 months of therapy, the distribution of percent weight-loss categories across treatment duration strata could not be robustly evaluated (sparse cells precluding a valid trend test). Nonetheless, the higher early-phase WEL scores suggest an initial motivational “lift” that may attenuate over time without structured support.

### 3.4. Correlations of WEL with Age and BMI

**Figure 1:**
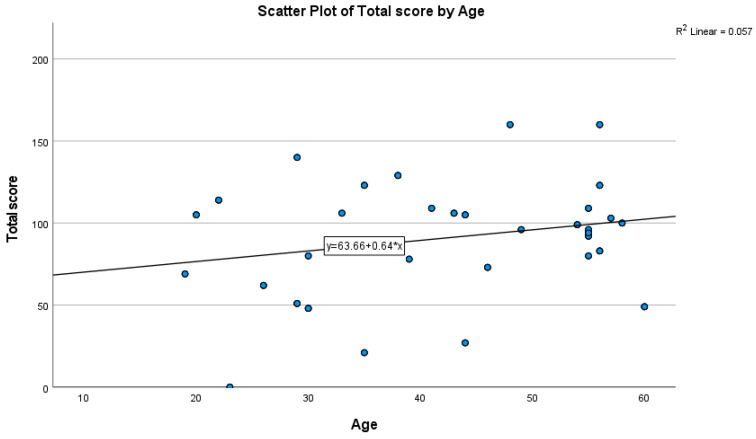
Scatterplot of WEL total versus age with fitted linear trend (positive slope).

Age showed a positive correlation with WEL total (r = 0.239, *p* = 0.009). In contrast, BMI did not (r = 0.004, *p* = 0.963), indicating that greater weight-control self-efficacy was associated with older age but not current BMI. Table 4, Figure 1 Qualitative findings (n = 15 interviews) **.** Four cross-cutting themes emerged: (1) perceived benefits and satisfaction; (2) side effect trajectory and self-management; (3) psychosocial impact and emotional well-being; and (4) motivation, adherence, and context; Table 5.

### 3.5. Four Cross-Cutting Themes Emerged (with Typical Variation)

**Perceived benefits and satisfaction.** Most participants reported high satisfaction with tirzepatide due to visible weight loss, improved appearance, and self-confidence. Many described greater energy and easier mobility/exercise (less breathlessness/fatigue). Several noted better sleep (faster sleep onset, deeper sleep). A minority reported slower-than-expected progress or limited change in activity.

**Side effect trajectory and self-management**. Initial GI-type symptoms (e.g., nausea, dizziness, headache, irritability) were common, generally mild to moderate and transient, sometimes recurring with dose increases. Most participants reported that side effects did not meaningfully interfere with daily activities beyond the first week; a few described brief activity adjustments (more rest, lighter schedules).

**Psychosocial impact and emotional well-being**. Many described improved mood, self-acceptance, and optimism; several linked these to weight changes and positive feedback from others. A subset reported no mental health change despite weight loss, underscoring heterogeneity in psychological response.

**Motivation, adherence, and context**. Common motivations included difficulty losing weight with traditional methods, health concerns (e.g., joint pain, diabetes risk), and appearance-related goals. Adherence facilitators included early weight changes, social encouragement, and insurance coverage; barriers included out-of-pocket costs and dose-related side effects. Many participants emphasized simple routines (timing injections, hydration, and smaller portions) as practical adherence supports.

### 3.6. Integration of Quantitative and Qualitative Findings

Early phase lift, later attenuation, financial context matters, and heterogeneous psychosocial response. These integrated insights suggest ongoing behavioral support and affordability measures alongside pharmacotherapy. A summary of key quantitative and qualitative integrations is provided. Early phase lift, later attenuation: Highest WEL scores in <1-month users, coupled with interview accounts of early motivation/optimism diminishing for some overtime, suggesting a need for ongoing behavioral support as treatment progresses. Financial context matters: Lower WEL among those paying out-of-pocket aligned with narratives about cost stress affecting confidence and adherence.

**Heterogeneous psychosocial response**: Quantitative differences by sex/employment echoed themes of social support/structure facilitating self-regulation, while qualitative heterogeneity in mood benefits cautions against assuming uniform psychological gains.

## 4. Discussion

This mixed-methods study adds real-world evidence to the tirzepatide literature by centering adults’ lived experiences alongside quantitative indicators of eating self-efficacy. Participants reported improvements across physical, emotional, and social domains, consistent with pharmacologic mechanisms and trial outcomes [2,3,4,8,9,12,13].

### Principal Findings in Context

Participants demonstrated generally strong weight-efficacy beliefs across social and affective situations, aligning with reports that GLP-1-based therapies can attenuate appetite and reward-related eating and support more mindful eating patterns [13]. Qualitative accounts in semaglutide users similarly describe reduced urges to eat under stress, suggesting a class effect that may facilitate adherence to lifestyle prescriptions. Notably, our item-level profile showed comparatively lower confidence for anxiety, depression, cravings, parties, and high-calorie availability, echoing evidence that hedonic cues can remain potent even with pharmacotherapy and that targeted behavioral strategies are needed in parallel [14]. Prior work has also observed that emotional-eating improvements are not universal under GLP-1 therapy [15], underscoring inter-individual variability and the value of adjunct behavioral support. Sociodemographic and access gradients were also analyzed.

Women reported higher self-efficacy than men, consistent with the literature showing stronger engagement with health-promoting behaviors among women in structured weight management [16,17]. Employment was associated with higher self-efficacy plausibly through greater routine, resources, or social structure that support health behaviors [18]. Insurance coverage patterns also mapped onto higher self-efficacy versus self-pay, aligning with research on financial toxicity and medication adherence: out-of-pocket costs can erode motivation and persistence [19]. Together, these findings point to modifiable system-level levers (coverage, counseling access) to bolster treatment success. Treatment exposure and prior attempts: Highest self-efficacy in the earliest exposure window may reflect a “honeymoon” period of optimism that attenuates without reinforcement, arguing for planned dose-titration support, side effect management, and periodic skill “boosters” to sustain gains [20,21]. Participants with prior diet-based attempts showed greater self-efficacy than those reporting fasting-only or activity-only approaches, mirroring evidence that structured, sustainable nutrition strategies better generalize to daily coping [22,23].

Patient-reported benefits beyond weight: Many participants reported improved sleep quality, energy, and mood, and findings consistent with data indicating that weight reduction and GLP-1 therapies can lead to better sleep (including OSA) and improved quality of life [24,25]. Still, variability was common: some reported unchanged activity or energy, highlighting the roles of baseline comorbidity, life demands, and the need for tailored behavioral activation [26]. Side effects were typically mild-to-moderate and transient, consistent with trial and real-world reports, yet dose escalations occasionally amplified symptoms, reinforcing the value of anticipatory guidance and pragmatic titration.

**Implications for practice and policy:**

- Pair tirzepatide with structured behavioral support targeting hedonic cues (parties, cravings, high-calorie availability) and negative affect (stress, loneliness).
- Plan adherence supports at the 1–6-month mark (digital check-ins, skills refreshers, dose-escalation side effect troubleshooting).
- Address affordability and coverage barriers to mitigate financial stress that may undermine persistence.
- Track patient-reported outcomes (sleep, mood, energy, social functioning) alongside weight and metabolic markers.

**Strengths and limitations.** Strengths include an explanatory sequential design, standardized self-efficacy measurement, and triangulation with qualitative interviews. Limitations include convenience sampling from a single region, one-group pre–post design (no control), reliance on self-report, and short-term follow-up; these limit causal inference and generalizability.

## 5. Conclusions

In community settings, adults using tirzepatide reported broadly positive experiences, higher eating self-efficacy, and perceived gains in sleep, energy, and mood alongside typically transient gastrointestinal effects. Variation by treatment duration and affordability highlights the importance of pairing pharmacotherapy with structured behavioral support and addressing coverage to sustain benefits.

### 5.1. Future Research

Key focus domains are as follows: (a) long-term studies designed to follow self-efficacy, adherence, and patient-reported outcomes (PROs) over at least 12 months; (b) practical trials of behavioral interventions that directly address stress reactivity and hedonic triggers through the context of oftirzepatide treatment; (c) equity-informed assessments of coverage models and their effects on persistence of treatment; and (d) studies aimed at mechanisms that connect changes in reward-based eating with changes in affect.

### 5.2. Data Sharing

De-identified quantitative data, the interview guide, and analysis code will be shared on reasonable requests to the corresponding author, subject to institutional approvals and confidentiality.

## Figures and Tables

**Table 1:** Baseline characteristics and tirzepatide use (N = 120).

Variable	Response	n (%)
**Gender**	Male	61 (50.8)
	Female	59 (49.2)
**Age (years), mean ± SD**	42 ± 13	
**Employment status**	Yes	77 (64.2)
	No	43 (35.8)
**Health insurance**	No insurance	42 (35.0)
	Government insurance	34 (28.3)
	Private insurance	23 (19.2)
	Employer-provided	21 (17.5)
Payment method for tirzepatide	Personal income	40 (33.3)
	Covered by insurance	35 (29.2)
	Partially covered by insurance	45 (37.5)
BMI (kg/m^2^), mean ± SD	—	25.74 ± 3.96
Duration of tirzepatide use	<1 month	27 (22.5)
	1–3 months	31 (25.8)
	3–6 months	45 (37.5)
	>6 months	17 (14.2)
**Previous weight-loss methods tried**	No	104 (86.7)
	Diet only	8 (6.7)
	Fasting	4 (3.3)
	Physical activities	4 (3.3)
Weight loss since starting tirzepatide	None	10 (8.3)
	<5% of initial weight	45 (37.5)
	5–10%	32 (26.7)
	>10%	33 (27.5)

Footnote: BMI = body mass index; SD = standard deviation. Percentages may not total 100% due to rounding.

**Table 2:** WEL item-level means and total score (higher = greater self-efficacy; scale 0–9).

Item	Mean ± SD
I can resist eating when I feel anxious (stressed).	4 ± 3
I can resist eating even when I have to say “No” to others.	5 ± 2
I can resist eating even when others pressure me to eat.	5 ± 2
I can resist eating when I feel tired.	5 ± 2
I can resist eating even when high-calorie foods are available.	4 ± 2
I can resist eating when I am angry.	5 ± 2
I can resist eating when I feel depressed.	4 ± 2
I cannot resist eating when I am at a party.	4 ± 2
I can resist eating even when I am happy and celebrating.	5 ± 2
I can resist eating when I feel bored.	5 ± 2
I can resist eating when I feel physically uncomfortable.	5 ± 2
I can resist eating even when traveling/away from home.	4 ± 2
I can resist eating even when watching TV.	5 ± 2
I can resist eating even when others around me are eating.	4 ± 2
I can resist eating even late at night.	5 ± 3
I can resist eating after a difficult day.	5 ± 2
I can resist eating when I feel lonely.	4 ± 2
I can resist eating when celebrating a special occasion.	5 ± 2
I can resist eating despite a strong craving for a specific food.	4 ± 2
I can resist eating when I feel sad.	4 ± 2
**Total WEL score**	**91 ± 34**

Negatively worded item: higher raw mean reflects greater difficulty in that context. Footnote: SD = standard deviation; response range 0 (“not confident”) to 9 (“very confident”).

**Table 3:** WEL total by demographic and treatment characteristics.

Variable	Category	Mean ± SD	95% CI (Mean)	*p*-Value
Gender	Male	87 ± 39	77.2 to 96.8	0.01 *
	Female	94 ± 28	86.9 to 101.1	
Employment	Yes	93 ± 39	84.3 to 101.7	0.048 *
	No	87 ± 22	80.4 to 93.6	
Health insurance	No insurance	99 ± 26	91.1 to 106.9	<0.001 *
	Government insurance	101 ± 28	91.6 to 110.4	
	Private insurance	71 ± 33	57.5 to 84.5	
	Employer-provided	80 ± 46	60.3 to 99.7	
Payment for tirzepatide	Personal income	84 ± 30	74.7 to 93.3	<0.001 *
	Covered by insurance	93 ± 37	80.7 to 105.3	
	Partially covered by insurance	94 ± 35	83.8 to 104.2	
Duration of use	<1 month	102 ± 35	88.8 to 115.2	<0.001 *
	1–3 months	89 ± 20	82.0 to 96.0	
	3–6 months	89 ± 35	78.8 to 99.2	
	>6 months	78 ± 46	56.1 to 99.9	
Previous attempts	No	92 ± 34	85.5 to 98.5	<0.001 *
	Diet only	99 ± 38	72.7 to 125.3	
	Fasting	68 ± 19	49.4 to 86.6	
	Physical activities	71 ± 50	22.0 to 120.0	
Weight loss since tirzepatide	None	92 ± 11	85.2 to 98.8	0.003 *
	<5%	101 ± 33	91.4 to 110.6	
	5–10%	83 ± 30	72.6 to 93.4	
	>10%	83 ± 41	69.0 to 97.0	

Differences (Welch 95% CIs): Female–Male = 7·0 (−5.1 to 19.1); Employed–Unemployed = 6.0 (−4.9 to 16.9). Analytic notes: Independent-samples *t*-tests (two groups) and one-way ANOVA (≥3 groups); * significant at *p* < 0.05. SD = standard deviation.

**Table 4:** Correlation of WEL total with age and BMI.

Variable	Pearson *r*	95% CI (Two-Tailed)	*p*-Value
Age	0.239	0.062 to 0.401	0.009
BMI	0.004	−0.175 to 0.183	0.963

Analytic note: Pearson correlation; significance at *p* < 0.05.

**Table 5:** Summary of patient-reported experiences (selected interview prompts, n = 15).

Interview Prompt	Summary of Participant Responses
Satisfaction with weight-loss results	Mostly satisfied/very satisfied (visible loss, confidence); a few slower or below expectations.
Physical activity changes	Often easier movement/exercise; less breathlessness/fatigue; some unchanged due to low baseline activity.
Sleep quality	Frequently improved (deeper, faster onset); some unchanged.
Energy levels	Commonly increased energy/reduced fatigue; some unchanged.
Mental health/self-confidence	Often improved mood and confidence; some unchanged/continued negative feelings.
Motivation to start	Difficulty losing weight, health issues, appearance, and clinician advice.
Emotional health overall	Greater self-acceptance/well-being for many; some unchanged.
Social interactions	Many reported more participation/encouragement; some reported no change.
Side effects (type/severity)	Early mild–moderate GI-type symptoms; occasional severe with dose increases; some none.
Impact on daily activities	Typically, the effects are minimal in the first week, with few reported temporary reductions in activity.

## Data Availability

The data presented in this study are available on request from the corresponding author due to privacy concerns.

## Supplementary Materials

The following supporting information can be downloaded at: https://www.mdpi.com/article/10.3390/healthcare13233102/s1, File S1: Semi-structured interview guide.
