# Supplementary material for: Exploring Adults’ Experiences with Tirzepatide for Weight Loss: A Mixed-Methods Study
Source: Healthcare (Basel). 2025 Nov 28;13(23):3102. doi: 10.3390/healthcare13233102 (PMC12691977; doi:10.3390/healthcare13233102)
Supplement: Supplementary file 1 [file healthcare-13-03102-s001.zip › healthcare-3919874-supplementary.pdf]

Supplementary File S1. Semi-Structured Interview Guide

Exploring Adults' Experiences with Tirzepatide for Weight Loss: A Mixed-Methods Study

Purpose. To elicit real-world experiences with tirzepatide for weight management, including motivations, routines, side-effects and dose titration, adherence facilitators/barriers, and perceived changes in appetite, sleep, mood, activity, and overall satisfaction. The interview complements quantitative findings (e.g., WEL scores) to inform mixed-methods integration.

A. Pre-Interview Checklist

• Confirm eligibility, preferred language (Arabic/English), and privacy for audio-recording.

• Re-confirm written informed consent; obtain verbal consent to audio-record.

• Explain confidentiality, right to skip any question or stop at any time, and that participation will not affect care.

• Remind participants not to share names or other direct identifiers during the interview.

• Have the adverse-event safety script available (see Section F).

• Note modality (in-person/teleconference) and presence of interpreter (if any).

B. Opening Script (read verbatim)

Thank you for meeting with me today. We are interested in understanding people's experiences using tirzepatide for weight management in everyday life. There are no right or wrong answers, please share what feels true for you. With your permission, I will audio-record so I don't miss anything. You can choose not to answer a question or stop at any time. This is not medical care and I cannot provide personal medical advice. If anything you share suggests an urgent health issue, I will pause and provide information on how to contact a clinician or emergency services. May I start the recording now?

C. Timeline Mapping (2–4 min)

Prompt: "Let's quickly map your tirzepatide journey." (use the grid below as a mental guide)

Period / Date	Dose (mg)	Duration	Notableeffects/side-effects	Weight/behavior changes

D. Core Domains, Questions, and Probes

1) Motivations & Goals
• What led you to consider starting tirzepatide?

• What goals mattered most (e.g., health, appearance, energy, specific events)?

• How did a clinician, family, or media information influence your decision?

Probes: Ask about prior attempts (diet/activity/other medications) and expectations at start.

2) Routines & Self-Management
• Walk me through how you fit tirzepatide into your weekly routine (timing, reminders).

• What self-management strategies help (hydration, smaller portions, trigger planning)?

• Any cultural, family, or work routines that interact with your regimen?

Probes: Probe: fasting periods, social/holiday meals, travel, night shifts, prayer/fasting schedules.

3) Side-Effects & Dose Titration
• What side-effects—if any—did you notice when starting or changing dose?

• How did you manage them (e.g., meal timing, hydration, dose adjustments)?

• How did you and your clinician decide when to increase, hold, or decrease dose?

Probes: Probe for trajectory (first week vs later), severity, persistence, and any impact on daily activities/work.

4) Adherence: Facilitators & Barriers (incl. Affordability/Access)
• What makes it easier for you to stay on track?

• What makes it harder (cost, supply, clinic access, stigma, family obligations)?

• How do insurance coverage or out-of-pocket costs influence your use?

Probes: Probe: pharmacy stockouts; appointment wait times; injection comfort; reminders; support from others.

5) Appetite & Eating in Everyday Contexts
• How, if at all, has your appetite or portion size changed?

• Tell me about situations where it's hardest to stick with your eating plan (stress, parties, cravings).

• How do you handle highly tempting foods at home, work, or social events?

Probes: Link to WEL constructs: negative affect, social pressure, food availability, evenings/TV, travel.

6) Sleep & Energy
• Have you noticed any changes in sleep quality or daytime energy?

• Any changes in snoring, restless sleep, or daytime fatigue?

Probes: Probe: timing of injections relative to sleep; naps; caffeine; exercise timing.

7) Mood & Emotional Well-Being
• Any changes in mood, confidence, or stress since starting?

• How do emotions (stress, sadness, boredom) affect your eating or routines now?

Probes: If low mood, irritability, or concerning symptoms arise, see Section F (safety escalation).

8) Physical Activity & Function
• Have you noticed changes in movement, activity, or daily function (stairs, walking, exercise)?

• Any changes in pain, breathlessness, or stamina?

Probes: Probe: how activity fits with appetite changes; any new or resumed activities.

9) Social & Work/Study Life
• How has tirzepatide affected social life, family roles, or work/study?

• What kinds of comments or support have you experienced from others?

Probes: Probe: stigma or encouragement; managing meals at work/school; travel requirements.

10) Satisfaction, Preferences, and Persistence
• Overall, how satisfied are you with tirzepatide so far? (0–10) Why?

• What would you change about your treatment plan or support?

• Do you plan to continue, pause, or stop? What would influence that decision?

Probes: Probe: balance of benefits/side-effects; affordability; long-term expectations.

11) Interruptions/Discontinuation (if applicable)
• Have you ever taken a break or stopped? What were the reasons?

• What happened during the break (weight, appetite, mood, sleep)?

Probes: Probe: how restarting felt; barriers to re-initiating.

12) Information Sources & Support Needs
• Where do you get information or support (clinic, pharmacist, online, friends/family)?

• What additional support would be most helpful?

Probes: Probe: preference for digital check-ins, group sessions, written guides, or clinician follow-ups.

E. Mixed-Methods Linkage Notes (for interviewers/analysts)
• If the participant completed the WEL, consider brief follow-ups on contexts where confidence was low (without reading items verbatim).

• Flag segments that illustrate: (a) early-phase motivational 'lift'; (b) later attenuation without support; (c) affordability constraints; (d) heterogeneous psychosocial responses (sleep, mood, energy).

• Capture concrete behavior changes (portion size, food choices, meal timing, social/holiday strategies) to triangulate with quantitative outcomes.

F. Participant Safety & Escalation Script
Read if concerning symptoms are reported (e.g., severe or persistent abdominal pain, repeated vomiting, signs of dehydration, allergic reaction; or serious mood concerns):

— "Thank you for sharing that. Because your safety is most important, I recommend you contact your clinician to discuss these symptoms. If you feel very unwell, please seek urgent care or emergency services. I can pause the interview now and provide contact details. Would you like to continue or stop here?"

If a participant expresses suicidal thoughts or intent, follow IRB-approved crisis procedures immediately and contact emergency services per site protocol.

G. Closing (read verbatim)
"Those are all my questions. Is there anything we didn't cover that you think is important to share? Thank you for your time and insights."

H. Interview Cover Sheet (to be completed by interviewer; do not record identifiers in audio)

- Participant ID (study code only): ________
- Interview date: ____/____/______ Start time: ____:____ End time: ____:____
- Modality (in-person / teleconference): __________ Language: __________
- Interpreter present? (Y/N): ____
- Current tirzepatide dose and duration (self-report): ___________________________
- Any safety escalations triggered? (Y/N) If yes, brief note: ____________________
